# Parallel Optimisation and Implementation of a Real-Time Back Projection (BP) Algorithm for SAR Based on FPGA

**DOI:** 10.3390/s22062292

**Published:** 2022-03-16

**Authors:** Yue Cao, Shuchen Guo, Shuai Jiang, Xuan Zhou, Xiaobei Wang, Yunhua Luo, Zhongjun Yu, Zhimin Zhang, Yunkai Deng

**Affiliations:** 1 Aerospace Information Research Institute, Chinese Academy of Sciences, Beijing 100094, China; laneruoyi@163.com (S.G.); jiangshuai@aircas.ac.cn (S.J.); zhouxuan@aircas.ac.cn (X.Z.); wangxb@aircas.ac.cn (X.W.); luoyh@aircas.ac.cn (Y.L.); yuzj@aircas.ac.cn (Z.Y.); zmzhang@mail.ie.ac.cn (Z.Z.); ykdeng@mail.ie.ac.cn (Y.D.); 2 School of Electronic, Electrical and Communication Engineering, University of Chinese Academy of Sciences, Beijing 101408, China

**Keywords:** synthetic aperture radar (SAR), back-projection algorithm (BP), real-time image processing, field-programmable gate array (FPGA)

## Abstract

This study conducts an in-depth evaluation of imaging algorithms and software and hardware architectures to meet the capability requirements of real-time image acquisition systems, such as spaceborne and airborne synthetic aperture radar (SAR) systems. By analysing the principles and models of SAR imaging, this research creatively puts forward the fully parallel processing architecture for the back projection (BP) algorithm based on Field-Programmable Gate Array (FPGA). The processing time consumption has significant advantages compared with existing methods. This article describes the BP imaging algorithm, which stands out with its high processing accuracy and two-dimensional decoupling of distance and azimuth, and analyses the algorithmic flow, operation, and storage requirements. The algorithm is divided into five core operations: range pulse compression, upsampling, oblique distance calculation, data reading, and phase accumulation. The architecture and optimisation of the algorithm are presented, and the optimisation methods are described in detail from the perspective of algorithm flow, fixed-point operation, parallel processing, and distributed storage. Next, the maximum resource utilisation rate of the hardware platform in this study is found to be more than 80%, the system power consumption is 21.073 W, and the processing time efficiency is better than designs with other FPGA, DSP, GPU, and CPU. Finally, the correctness of the processing results is verified using actual data. The experimental results showed that 1.1 s were required to generate an image with a size of 900 × 900 pixels at a 200 MHz clock rate. This technology can solve the multi-mode, multi-resolution, and multi-geometry signal processing problems in an integrated manner, thus laying a foundation for the development of a new, high-performance, SAR system for real-time imaging processing.

## 1. Introduction

In the past decade, advances in digital and microwave technologies have led to the leap-forward development of synthetic aperture radar (SAR) with significant improvements in the device capability, system design, and processing algorithm. However, the SAR image generation and information processing link cannot match the SAR capability improvement to achieve the most efficient remote sensing information acquisition capability. The traditional remote sensing, SAR information acquisition process comprises the following: planning the platform flight routes and radar operation modes, collecting and recording radar echo data from the ground area using SAR radar, returning, copying, or uninstalling echo data, importing the data to ground high-performance computing devices, processing data with an algorithm, achieving the result, and forwarding the results to the users. It is observed that the traditional SAR remote sensing information acquisition process is time-consuming with many nodes and poor flexibility that cannot perform high-efficiency remote sensing detection of time-sensitive targets. To solve these problems, the real-time data processing capabilities should be enhanced to synchronise platform flight, data processing, user reception, and realise the real-time changes of platform flight routes or working modes according to user feedback to obtain more targeted real-time remote sensing results. Therefore, in-depth research on SAR real-time processing technology has far-reaching significance and great application value. To improve the SAR’s real-time processing capability and generated image quality, it is necessary to conduct in-depth research on imaging algorithms and hardware and software architectures [1,2,3,4].

In terms of algorithms, the rapid development of antenna technology has made its applications more and more versatile [5,6]; meanwhile, new mechanisms of SAR have been emerging. Consequently, the application platform from airborne and spaceborne was expanded to the foundation and on-board, the operational mode was upgraded from the stripe, bunching, and scanning to the tops, mosaic, and multimodal types, and the imaging geometry was extended to include side view, strabismus, large strabismus, and circular trail. Given the application requirements of multi-platform, multiple modes, and multiple geometries, imaging algorithms are required to have uniform and general characteristics. Currently, imaging algorithms can be divided into two categories: frequency-domain and time-domain algorithms. Common frequency-domain imaging algorithms include the range doppler (R-D), chirp scaling (CS), and wavenumber-domain (W-K) algorithms. These algorithms usually need to decouple the echo signal in range and azimuthal directions. There will be many approximations and assumptions in the process of decoupling, leading to errors in the imaging results. Moreover, the decoupling calculation at different flight paths, working modes, and geometric configurations are also very different, and the processing cannot be unified [7,8,9]. The time-domain imaging algorithm is the back-projection (BP) algorithm. Instead of pulse accumulation in the signal domain, the algorithm projects the echo data of each pulse to the image domain through backward projection in a successive manner and then accumulates energy coherently in the image domain. With the accumulation of energy, the image resolution gradually improves until the maximum resolution is obtained. Because there is no need for synthetic aperture signal accumulation, theoretically, imaging with the BP algorithm can yield outputs without delays. At the same time, because BP is based on the actual motion trajectory of the platform, it is not an approximation algorithm, and can be theoretically applied to any platform, track, geometric configuration, and imaging mode. This algorithm is the focus of this study [10,11,12,13,14,15,16,17].

In terms of hardware and software architectures, an increasing number of processing architectures and devices have been designed and applied to the field of real-time signal processing in recent years. The field-programmable gate array (FPGA) shows obvious advantages in many signal processing schemes by its full programmability, parallel processing, and low-power consumption [18], and the simultaneous development of high-level language synthesis technology in recent years has greatly improved the algorithmic aspect of FPGA programming [19]. The BP algorithm involves a considerable amount of computation calculations. Among these, the operation process involves a large amount of vector multiplication and read–write memory. However, the fully programmable and parallel multi-pipeline features of FPGA can adapt to the BP algorithm and can achieve an efficient structure for the algorithm [20,21,22]. Nowadays, research work targeting the BP algorithm and using FPGA to implement the algorithm has become noticeable. For example, three interpolation methods have been proposed in [23] to reduce the computational complexity of the interpolation part of the algorithm. In [24], the OpenCL framework has been used to implement fixed-point data processing in System on Chip (SoC) to reduce computing resources and improve clock performance. In [25], the BP designed by the logic part of SoC has been used to deal with Intelligent Property (IP). The IP can be flexibly deployed on a unit chip or multiple chips repeatedly. However, the above design still needs to be further improved in processing time efficiency.

In summary, this study designed an efficient architecture for the BP algorithm based on the FPGA processor, completed the SAR real-time imaging processing on a single chip, and laid a technical foundation for subsequent, complex, high-performance airborne and spaceborne real-time processing systems.

## 2. Principle of the BP Algorithm

**Figure 1:**
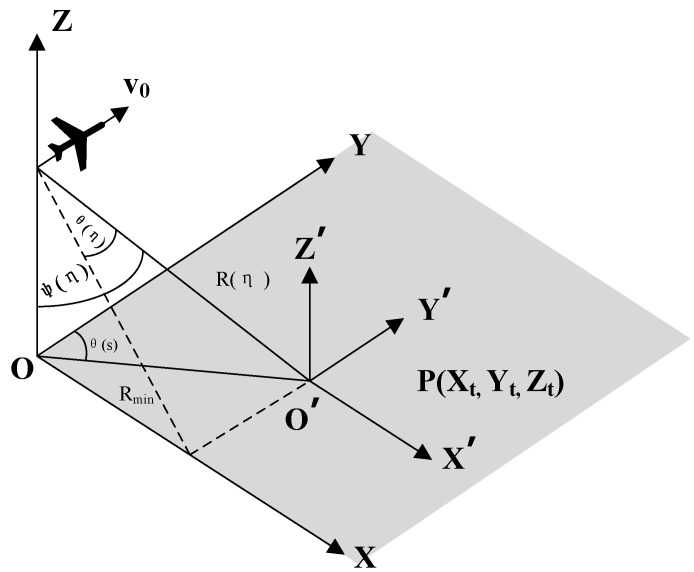
Airborne SAR signal model.

The airborne imaging model is shown in Figure 1. The linear airborne speed of the radar along the plane is *υ*_0_, and the northeast sky Cartesian coordinate system OXYZ is established. To better describe the target, the reference coordinate system O’X’Y’Z’ is established, in which the target’s coordinate is P(X*_t_*, Y*_t_*, Z*_t_*). R(*η*) represents the oblique distance from the radar platform to the target point at each azimuth time; R_min_ is the minimum oblique distance between the radar and the target point; θ(*η*) represents the viewing angle between the radar and the target at each azimuth moment; ψ(*η*) represents the pitch angle between the radar and the target at each azimuth time; and θ(*s*) is the azimuth angle between the radar and the target.

Based on the geometric relationship illustrated in Figure 1, the oblique distance from the radar platform to the target point R(*η*) at time *t* can be calculated as:

(1) $$R(\eta )\;=\;\sqrt{{\left[x(\eta )-X_{t}\right]}^{2}+{\left[y(\eta )-Y_{t}\right]}^{2}+{\left[z(\eta )-Z_{t}\right]}^{2}}$$

where (*x*(*η*)*, y*(*η*)*, z*(*η*)) is the trajectory of the aircraft platform, and (X*_t_*, Y*_t_*, Z*_t_*) represents the spatial position coordinates of the target P at time *t*.

Suppose the radar transmits the linear frequency modulation signal, and the echo signal is obtained after modulation and delay. After demodulation, the echo signal *s*(*τ*, *η*) of the target point can be expressed as:

(2) $$s(\tau ,\eta )=W_{r}[\tau -2R(\eta )/C]W_{a}(\eta -\eta_{c})*\exp[-j4\pi f_{0}R(\eta )/C]*\exp\{j\pi K_{r}{\left[\tau -2R(\eta )/C\right]}^{2}\}$$

where *f*_0_ is the carrier frequency of the transmitted pulse, *τ* represents the distance time, *η* represents the azimuth time, K*_r_* is the distance modulation frequency, and *η* represents the beam centre offset time. The echo data is transformed into the distance frequency domain, and expressed as S(*f_τ_*, *η*) as shown below:

(3) $$S(f_{\tau },\eta )=W_{r}(f_{\tau })W_{a}(\eta -\eta_{c})*\exp(-j\frac{4\pi (f_{0}+f_{\tau })R(\eta )}{c})*\exp(-j\frac{\pi f_{\tau }^{2}}{k_{r}})$$

Next, *s_rc_*(*τ*, *η*) is obtained from range pulse compression as below:

(4) $$s_{rc}(\tau ,\eta )=\mathrm{IFFT}\{S(f_{\tau },\eta )H(f_{\tau })\}$$

where $H(f_{\tau })=\exp(j\pi \frac{f_{\tau }^{2}}{k_{r}})$.

As a result, the radar echo pulse compression signal *s_rc_*(*τ*, *η*) is obtained, and the imaging result of the BP algorithm in aircraft motion can be calculated by geometric mapping.

**Figure 2:**
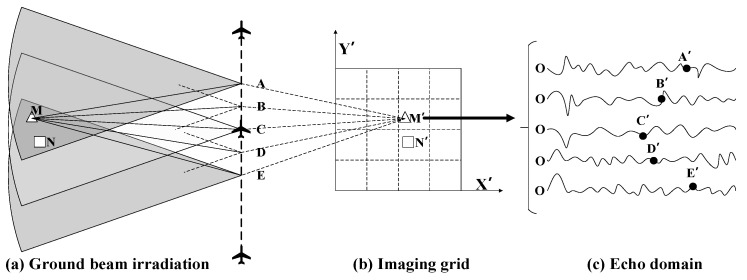
Working principle of the back-projection (BP) algorithm.

The actual working scenario of the simulated aircraft is as follows. The radar is radiated through the left side of the aircraft and transmits and receives echoes at the waypoints ABCDE (Figure 2). There are two targets M and N in the irradiation area. The imaging grid in the (X, Y) rectangular coordinate system is established, and M’ and N’ are the projections of the target in the imaging grid, and the route points A to E also represent the position of the antenna phase centre in the imaging process. The process of obtaining M’ by imaging M is equivalent to calculating the phase component from each pulse to the Mth point according to the distance between the antenna phase centre and the Mth point. The sum of phase components is the value of the Mth’ point, and the interception method involves the multiplication of the corresponding range gate data by the phase weight of the data in the echo domain. For example, the aircraft antenna centre is at point A, with the distance from the aircraft to the target M being denoted as AM. The phase component of the projection point M’ can be obtained as: $S\left(2\bar{\mathrm{AM}}/C,\eta_{a};M\right)$, where AM = AM’, *η_a_* is the azimuth moment corresponding to the antenna phase centre at point A, and the corresponding position of the echo data domain is A’. By analogy, the phase value of point M can be obtained at the phase centre of other antennas.

**Figure 3:**
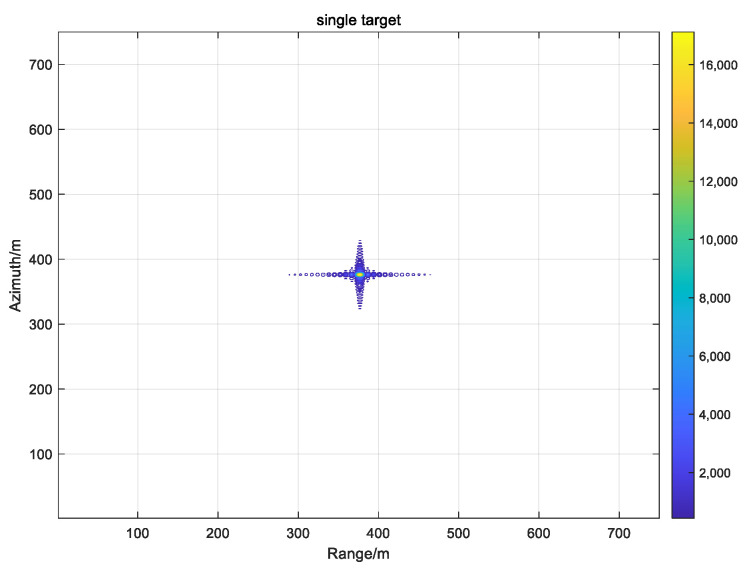
Single point simulation result.

By accumulating coherently the phase value of point M at the phase centre moment of each antenna, the imaging projection result of point M (point M’), can be obtained. The values are as follows:

(5) $$I(x,y)=\int {}_{\eta_{a}}s_{rc}(\tau_{r},\eta_{a};M)\exp\left[j\frac{4\pi f_{c}}{c}R_{M}(\eta_{a})\right]d\eta_{a}$$

where *R*_M_ is the distance from the centre of the antenna phase to point M. The phases of each pulse are extracted and accumulated separately so that the pixels on the imaging grid match exactly the real position of the antenna’s phase centre. Therefore, the calculation of the distance from the antenna phase centre to the target is a key part of the BP imaging algorithm. Theoretically, this method can be used to obtain high-precision SAR images without approximation, and the algorithm can be applied to high-precision imaging of complex geometric configurations, such as strabismus and circular trace. In practical engineering, the distance between the imaging grid and the phase centre of the antenna may not correspond to the data in the echo domain as integer multiples, and interpolation operation is required. The interpolation of multiple, interpolation functions and the calculation accuracy will directly affect the quality of the imaging. The simulation process is carried out with a point target as an example, and the results are depicted in Figure 3 as follows [26].

The results of the analysis of the target azimuthal metrics at the corresponding point targets for different numbers of pulse accumulations are shown in the table below.

It can be observed from Table 1 that the greater the number of pulse accumulations in the BP algorithm the smaller the IRW. Meanwhile, the PSLR and ISLR are almost constant, which is the difference between the time domain algorithms and frequency domain algorithms in the processing results.

Table 1 Results of the analysis of the target azimuth metrics of the point targets.

**Figure 4:**
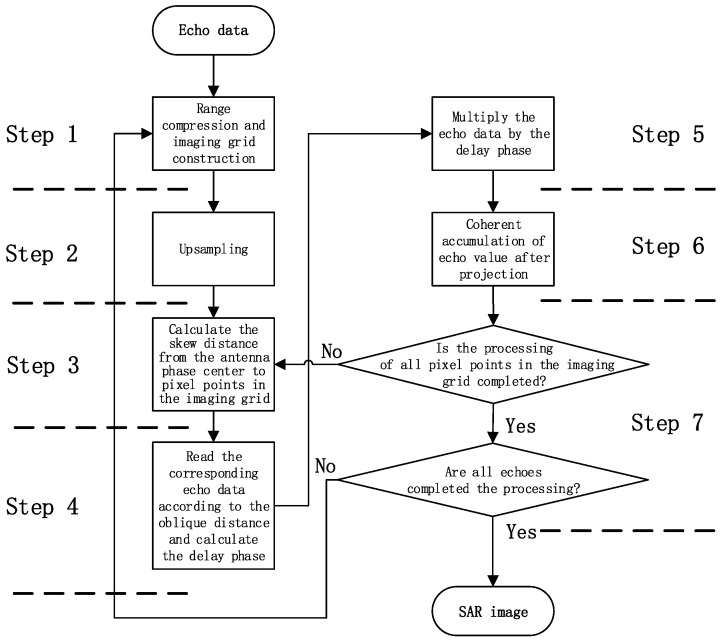
BP flow chart of the imaging algorithm.

Based on the above analysis, the flow chart of the BP algorithm is as Figure 4:

The main steps are as follows:

1. Step 1: Range compression is performed on the echo, and an imaging grid is constructed according to the resolution requirements (corresponding to the value of *s_rc_* and the coordinate system O’X’Y’Z’);
2. Step 2: Upsampling is performed on the data that has completed the range direction compression according to the imaging grid (aim to improve the accuracy of (*τ*,*η*) in *s_rc_*);
3. Step 3: Calculate the distance from the antenna’s phase centre to pixel points in the imaging grid (corresponding to the calculation $R_{M}(\eta_{a})$ in Equation (5);
4. Step 4: Read the corresponding echo data according to the distance and calculate the echo delay (corresponding to the calculation $s_{rc}(\tau_{r},\eta_{a};M)$ and $\exp\left[j\frac{4\pi f_{c}}{c}R_{M}(\eta_{a})\right]$ in Equation (5);
5. Step 5: Multiply the echo data according to the phase information;
6. Step 6: Coherent accumulation of echo value after projection (corresponding to the calculation of Equation (5);
7. Step 7: Repeat steps 1 to 6 for the subsequent echo until all pixels of the imaging network and all echoes are processed.

The computation of the BP imaging algorithm mainly focuses on range compression in step 1 and distance calculation in step 3, while the memory read–write operation mainly focuses on data read–write in the echo domain in step 4 and coherent accumulation of echo value in step 6. In step 1, the operation of range pulse compression mainly includes multiplication, addition, and division of complex numbers, and distance calculation mainly involves multiplication and addition of complex vector numbers. Memory reads and writes are large-scale discontinuous address reads and writes. The system processing parameters are set as follows: azimuth direction of echo data is A, range direction is R, and the size of imaging grid is N × N. Therefore:

- Range compression requires A × R × 2log_2_R + A × R times of the complex multiplication and addition;
- Distance calculation requires 5 × A × N × N times of complex multiplication and addition;
- Read-write echo domain data requires A × N times of memory reads and A × N × N times of memory writes;
- Coherent accumulation of echo value requires A × N × N times of memory data reads and A × N × N times of memory writes.

**Figure 5:**
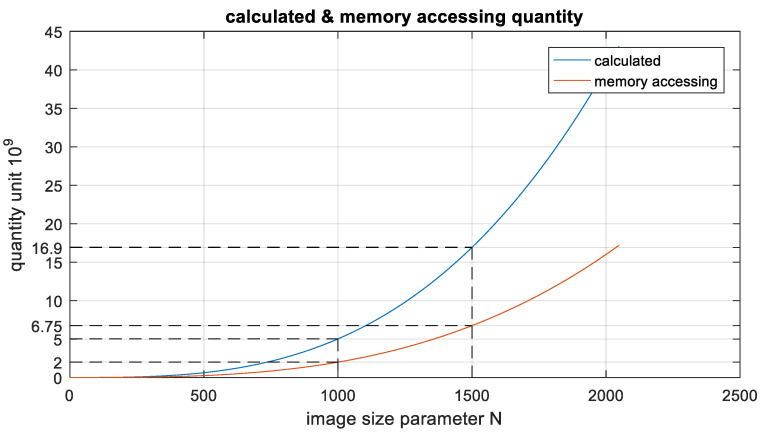
Plot of the quantity unit as a function of the image size parameter N for the calculated quantity and the number of memory reads and writes.

In actual engineering, the azimuth parameter A of the echo data is larger than the parameter N of the imaging grid. It can be observed from the above that the computational complexity of the BP imaging algorithm is mainly concentrated in the distance calculation part, the computational complexity of the algorithm has reached O(*N*^3^), and the number of memory reads and writes is also proportional to *N*^3^. The relationship between these three is shown in the Figure 5.

It can be observed from the above figure that when SAR images with the size of 1000 × 1000 pixels are generated, approximately 5 G times of multiple accumulations, and 2 G times of memory reads and writes are required. When the image size becomes 1500 × 1500, the times of multiple accumulations and memory reads and writes increase to 16.9 G times and 6.75 G times, respectively. In the airborne platform, the pulse repetition rate (PRF) can exceed 4 K; thus, the amount of computation and memory reads and writes per unit time is huge. The Xilinx XC7VX690T FPGA processing chip is selected in this study. Based on this chip, real-time imaging with a pixel size of 1000 × 1000 can be realised.

In summary, although the BP algorithm does not apply any approximation to the signal processing imaging process, it can accurately eliminate the coupling between range and azimuth, and the obtained image does not need to be processed for geometric correction. However, owing to the huge amount of computation, the application of the BP algorithm in real-time imaging of large-scale scenes is limited. Therefore, finding ways to optimise the BP algorithm for efficient implementation by small-scale processing hardware has important research significance.

## 3. Algorithmic Implementation for Architectural Design and Optimisation

Advanced processing architecture design and optimisation are needed to efficiently implement the BP algorithm. The processing architecture should be fully integrated with the FPGA structure and the characteristics of the algorithmic process. Based on the above, this study conducts in-depth research on the implementation of the algorithm architecture from the perspective of the (a) optimisation process [27], (b) fixed-point operation optimisation [28], (c) parallel processing optimisation [29], and (d) distributed storage [30].

### 3.1. Algorithmic Flow Optimisation

The algorithmic flow optimisation mainly includes adjusting the processing steps to adapt the algorithm to the FPGA processing hardware and the pipelined design to improve the computational throughput. Figure 3 is the most original flow diagram of the BP algorithm based on PC. This flow first stores data in PC memory and then processes it serially. The direct deployment of this flow to FPGA will lead to extremely low-calculation efficiency mainly owing to the following three reasons and optimisation methods:

1. There are iterations and branch jumps in the main process. Iteration and branch jumps will reduce the efficiency of FPGA calculations and cannot realise pipelining. The optimisation method involves the expansion of the iteration cycle and the data-flow processing on the top of the code architecture so that the main process used to achieve flow processing is highly suitable for accelerated implementation on FPGA hardware;
2. Dynamic random-access-memory (DDR) access exists. Memory access will reduce the processing flow and memory interaction will increase the processing delay. The processing flow based on FPGA can directly send data to the processing module for calculation instead of accessing the memory frequently to transfer data for processing;
3. Redundant computation exists. The original BP algorithm usually uses the frequency domain method to directly interpolate data by eight-fold in the upsampling part. It then selects the corresponding result after interpolation when reading echo domain data in step 4. In this step, there are usually one or two interpolation points. Thus, there are 3/4 or even 7/8 redundant calculations in the upsampling part. The optimisation method will then select the sinc (x) function (SINC) interpolation algorithm in the time domain to accurately calculate and generate the echo-domain data required in step 4, rather than interpolating the data first and then reading and storing the data.

**Figure 6:**
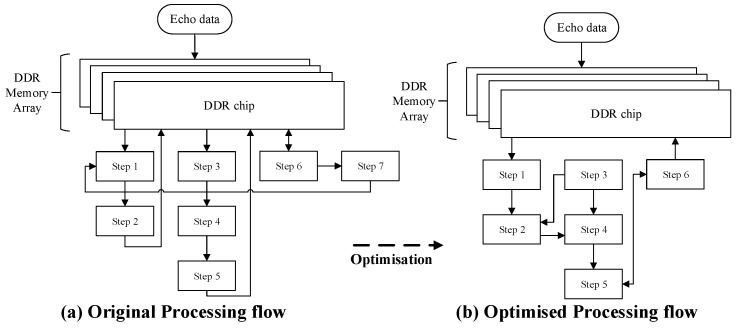
Algorithmic flow optimisation.

In summary, the optimised result of the algorithm flow is shown in Figure 6. Step 1 reads the data in DDR for pulse compression and coordinate system establishment; step 2 receives the processing result of step 1, as well as the distance calculation result of step 3 for parallel upsamplings; step 4 receives the processing result of step 3 and calculates the delay phase at the same time; step 5 multiplies the echo data and delay phase; and step 6 connects DDR and accumulates the processing result of step 5 with the data in DDR. The loop of these six steps continues to cycle until the data processing is finished and a clear SAR image is obtained. Compared with the original process, the optimised process has three advantages: first, the original processing flow interacts with DDR memory many times, while the optimised post-processing flow is highly pipelined leaving only two channels to access the memory; second, the upsampling in step 2 is precisely controlled by the location of imaging grid from step 3, which improves the pertinence of computing power and removes redundant calculations; third, by removing step 7, the whole process has no branch jumps, resulting in more efficient processing.

### 3.2. Fixed-Point Operation Optimisation

**Figure 7:**
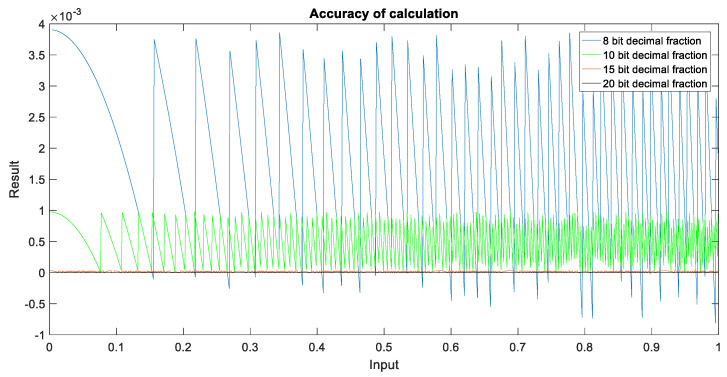
SINC interpolation kernel operation accuracy.

The processing accuracy is an important factor affecting the occupied resources and output results. During the operation, the accuracy of the SINC interpolation in the upsampling step 2 and the numerical range of the echo accumulation in step 6 should be fully considered, and the impact on the memory capacity and bandwidth should be considered when storing. The analysis of the impact of the two is as Figure 7.

It can be observed from the above figure that for SINC interpolation operation, the 8-bit fixed-point decimal precision is approximately 10^−3^, the 10-bit fixed-point decimal precision is approximately 10^−4^, the 15-bit fixed-point decimal precision is approximately 10^−6^, and the 20-bit fixed-point decimal precision is approximately 10^−7^. Regarding the dynamics that can be represented by data, the maximum dynamics operations of the 8-bit fixed-point is 256, 11-bit is 2048, 16-bit is 65,536, 20-bit is 1,048,576, and single-precision floating point is 6.8 × 10^38^. In the actual project, an accuracy of 10^−6^ has met the calculation accuracy requirements, so the 15-bit fixed-point decimal is finally selected for calculation.

**Figure 8:**
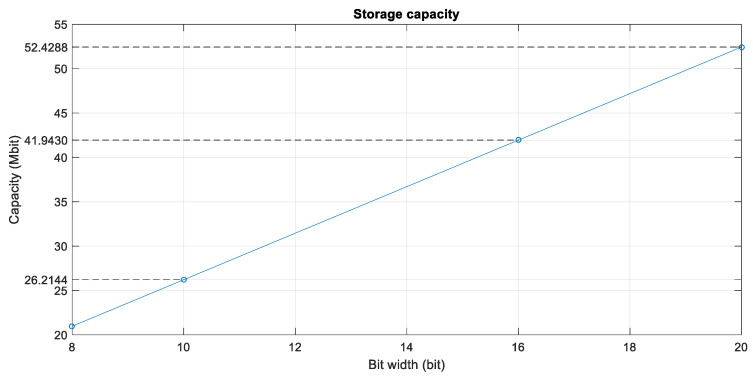
Variation of storage capacity as a function of the data-bit width.

The relationship between the storage capacity and bit width calculated based on the optimised processing flow is as shown in the following Figure 8.

The 10-bit memory requirement is 26.2144 Mb, the 16-bit memory requirement is 41.9430 Mb, and the 20-bit memory requirement is 52.4288 Mb. Different precisions of the same operation consume different processing resources. Using the range pulse compression in algorithm step 1 as an example, the processing resource consumptions are compared [31] in the following Table 2.

It can be observed from Table 2 that as the bit width increases, the accuracy and dynamics increase, but at the same time, the requirements for memory and processing resources increase. Therefore, it is necessary to achieve a compromise between the processing results and the occupied resources. The generated point target was simulated by MATLAB with various precision processing results and the analysis is summarised below.

**Figure 9:**
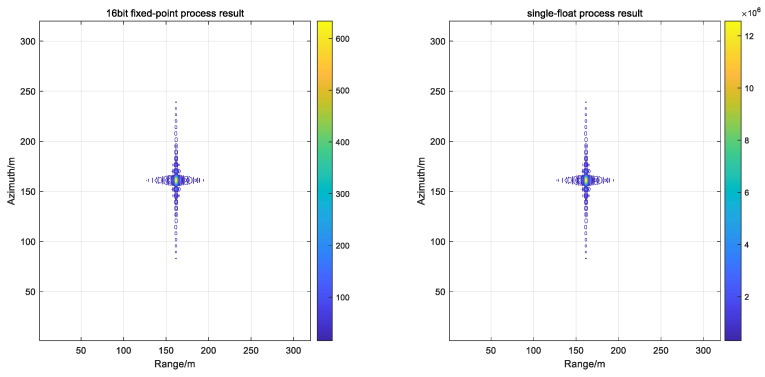
Bit-wise 16 bit vs. single-precision processing results.

The Figure 9 on the left shows the result of the 16-bit fixed-point processing, and the figure on the right shows the result of the single-precision floating-point processing. After numerical analysis, the focus metrics were observed to be the same for the PSLR, ISLR and IRW. However, the amplitude values were different. In practical applications, the image output is 8 bit-quantized, therefore, the amplitude variation has a negligible impact on the result.

**Figure 10:**
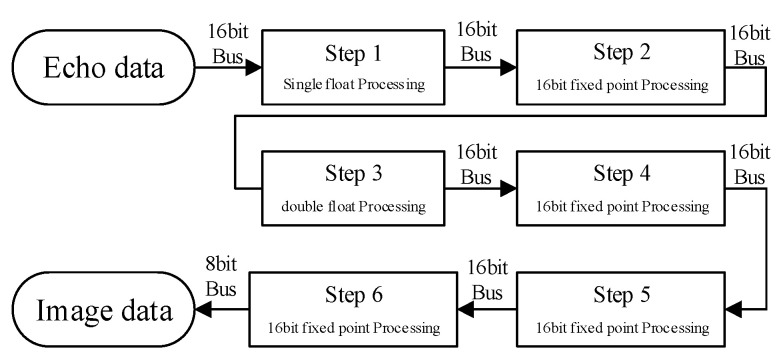
Bit-width design in the processing flow.

To obtain the optimal processing results, the processing accuracy needs to be combined with fixed-point and floating-point starting from the simulation results and combining the Xilinx XC7VX690T FPGA chip resources [32]. The data input is buffered into the DDR by a 16-bit fixed-point, and the range compression by floating-point processing. After processing, 16-bit, fixed-point quantisation is performed, and the results are stored in RAM. Distance is calculated by a double floating point. SINC interpolation by 16-bit fixed-point. The value coherent accumulation adopts 16-bit fixed-point, and the final result adopts 8-bit fixed-point output. The entire processing bit width is designed as shown in the Figure 10.

### 3.3. Parallel Processing

**Figure 11:**
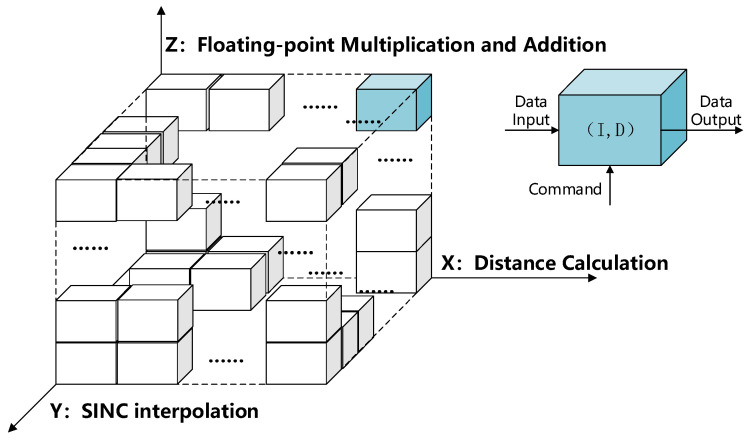
Schematic diagram explaining parallel data processing.

To improve the system processing capability and shorten the processing time, the BP algorithm needs to be parallelised. Parallel algorithmic processing is implemented with single-instruction, multiple-data (SIMD), and multiple-instruction, multiple-data (MIMD) processing models in three dimensions. The first dimension is distance calculation. Each distance projection of the BP algorithm is completely independent. Combined with the computing resources of each path, 16 paths are designed for parallel execution of this dimension. The second dimension is SINC interpolation [33]. The optimised flow chart shows that after the distance calculation, the echo needs to be upsampled. The upsampling uses an 8-point interpolation core. Thus, eight paths are designed for parallel execution. The third dimension is floating-point multiplication and addition [34]. More than eight blocking operation cycles are required for each high-precision data multiplication and addition, which makes the entire process unable to achieve efficient pipeline processing. Therefore, it is necessary to improve the throughput through parallel processing by adopting the eight-path parallel execution. In summary, the three dimensions perform sixteen-path, eight-path, and eight-path parallel processing, respectively. There are 1024 processing units in each clock cycle. The first and the second dimensions are MIMD, and the third dimension is SIMD. The schematic diagram of parallel processing is as Figure 11.

**Figure 12:**
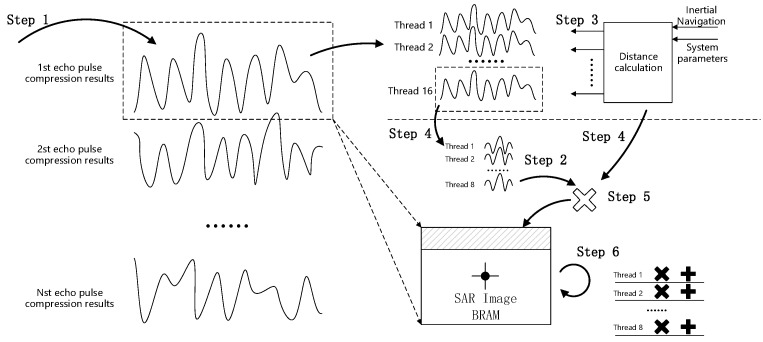
Parallel implementation process.

Each box in the figure above represents a processing unit, which has an independent clock and an input/output interface, and can flexibly adjust the operating rate, input data, and parameters. The three-dimensional processing model was applied to the BP algorithmic flow to form the following processing and control flow as Figure 12.

The flow of calculation can be summarised as follows. Step 3 controls step 4 to pass the processing result of step 1 to step 2; step 5 receives and processes the output of both step 2 and step 4, and then passes it to step 6. The data processing method is described in detail as follows:

Step 1 receives echo data while performing range pulse compression on the echo data, and makes 16 copies of the compressed data to prepare for subsequent parallel calculation; step 4 receives the inertial navigation information and system parameters in 16 parallel threads to calculate the slant range by passing the calculated results to steps 3 and 5; step 3 receives the distance information, indexing each range pulse pressure result and exporting the outputs to step 2; step 2 receives the data and starts processing in eight parallel threads; step 5 receives the data from steps 2 and 4, and processes with high precision multiplication, writing the result to the Block RAM (BRAM); step 6 reads the BRAM data and initiates the multiple accumulation operation of eight parallel threads. The three-dimensional processing model corresponds to steps 4, 2, and 6, respectively. After the parallel optimisation, the data throughput rate can reach 200 Gsps, which meets the requirements of the BP algorithm for real-time imaging data calculation of the throughput rate.

### 3.4. Distributed Storage

The BP algorithm not only requires huge computing ability but also imposes strict requirements on the throughput rate of data access. There are a lot of data fetches in steps 4 and 6. To meet this access bandwidth requirement, a distributed storage design based on FPGA is required. The FPGA processing platform has three flexibly configurable storage resources, namely register (reg), static RAM (SRAM), and dynamic RAM (DDR). The structures and characteristics of the three are different. The specific indicators are as follows Table 3.

**Figure 13:**
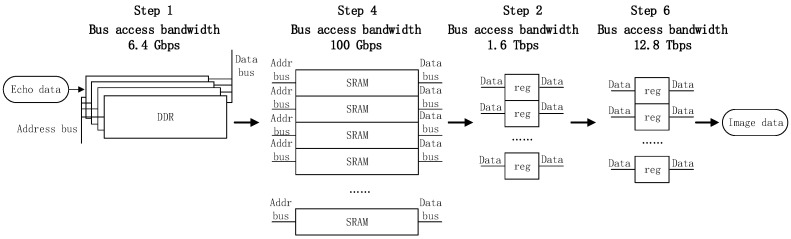
Distributed storage schematic.

Combined with parallelised design, the advantages of three types of memory are fully utilised to realise high-throughput computing. The first parallel dimension 16-way calculation is implemented by SRAM, the single-way port width is 32-bit, the interface speed is 200 MHz, and the bus speed is 100 Gbps; the second parallel SINC interpolation eight-way calculation is implemented by register storage, and the single-way port width is 64. The bit-interface speed is 200 MHz, and the bus speed is 1.6 Tbps; the third parallel, high-precision, multiply–add, eight-way calculation is implemented by register storage. The single-channel port width is a 64-bit interface operating at the frequency of 200 MHz, and the bus speed is 12.8 Tbps. The dynamic RAM buffer is added in the echo input and image result output, thus realising the isolation of the processing clock and data flow rate and improving the running speed of the entire calculation process. Based on the above design, the throughput of ultra-high data rate in the operation process is realised as Figure 13.

Step 1 uses a storage matrix composed of DDR particles, which shares a set of data and address buses, so the access speed is slow, but the capacity is large; step 4 uses a storage matrix composed of SRAM, wherein each of which has an independent address and data bus, coupled with the device characteristics of SRAM; thus, the access rate of the matrix is faster; steps 2 and 6 use registers to form a storage matrix, wherein each element of the matrix has only data bus and no address bus. Thus, it can be accessed in full parallel mode fast. To meet the real-time processing of the full-process BP algorithm, a distributed storage technology combining multiple storage devices is designed [35].

## 4. Integrated Design of Software and Hardware of Imaging Processing System Based on FPGA

**Figure 14:**
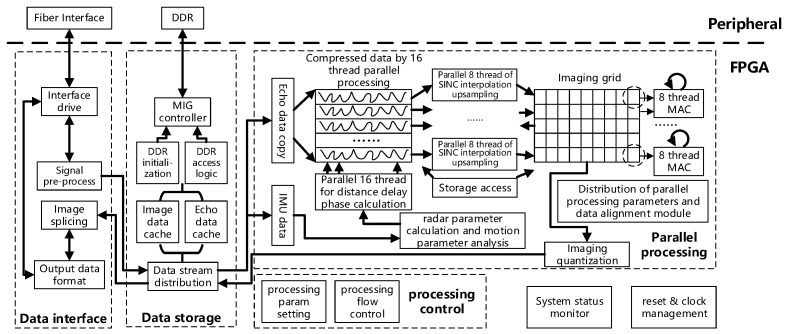
Block diagram of synthetic aperture radar (SAR) real-time imaging system.

This study proposes a block diagram of FPGA-based, SAR, real-time BP software and hardware integration scheme, as shown in the Figure 14. Data are input to FPGA through an optical fiber interface, and FPGA completes single-chip SAR imaging through four core processing units: data interface, DDR data storage, processing control, and parallel data processing.

The data interface unit includes the interface driver module, signal pre-processing module, image splicing module, and output data format module, which mainly completes input and output interface and format arrangements. The data storage unit consists of the MIG controller, DDR initialisation, DDR access logic, image data cache, echo data cache, and data stream distribution modules, which mainly complete the large-capacity and low-speed data buffer storage related to DDR. The processing control unit includes the processing parameter setting and processing flow control module, which mainly completes the top-level resource scheduling and control of the entire processing flow and the initialisation parameter configuration of each module. The parallel processing module is the core of the system. Data through data stream distribution module separate into echo data and IMU data, IMU data used for radar and motion parameter calculation. The calculation result is sent to parallel distance and delay phase computing modules, meanwhile, the echo data is copied 16 times for subsequent processing. In parallel, each copied data (among the 16 copies) of range compression to be processed is combined with the above distance and delay phase calculated results, and then the output is sent to the eight-thread parallel SINC interpolation upsampling module for calculation. The calculated results are sent to the imaging grid. Every data entering into the imaging grid is subjected to parallel, eight-thread multiplication accumulation calculation (MAC). After all the sampling data is processed, the data in the imaging grid is the image data, and the image data is output according to eight-bit quantisation. In this system, there are computing and storage modules related to the algorithmic software, system status monitoring, and reset and clock management modules related to hardware.

## 5. System Verification and Analysis

**Figure 15:**
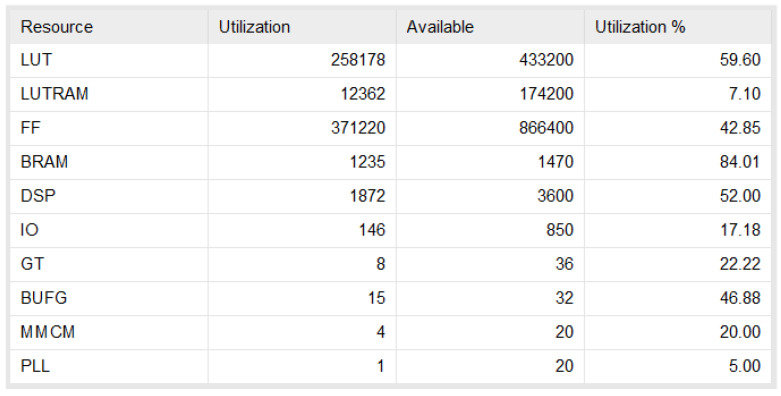
BP algorithm resource analysis.

The time-domain BP algorithm for SAR imaging is implemented in a Xilinx XC7VX690T FPGA chip. The development board contains one unit of XC7VX690T FPGA and an external 16 GB DDR3, whose clock speed is 1333 MHz. The bus is 64-bit wide. The FPGA has a clock speed of 200 MHz. In all, 16 threads perform phase calculations. If the input data is 16 bits, the maximum data input rate is therefore 51.2 Gbps. The resource usage is analysed in the Vivado development environment of Xilinx Inc. and the screenshot of the Vivado analysis result is shown in Figure 15 below.

**Figure 16:**
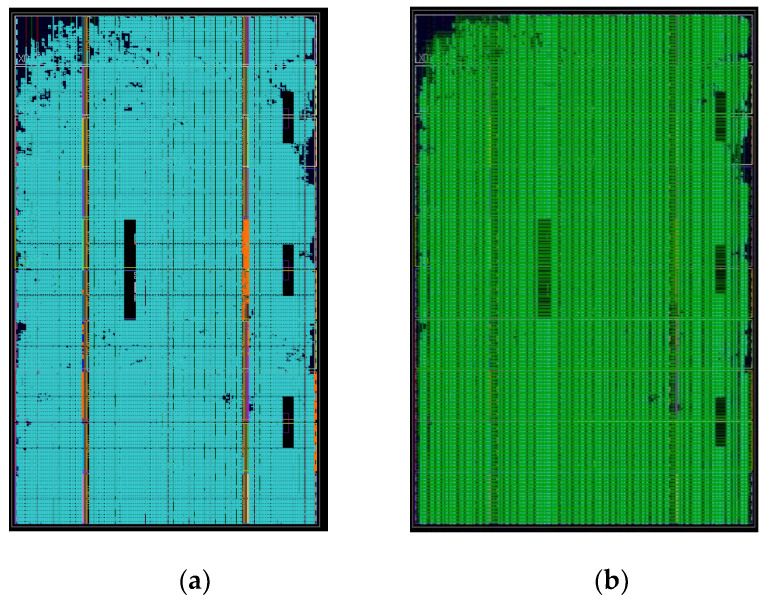
Resource layout and routing diagram. (**a**) Resource layout. (**b**) Resource routing diagram.

It can be observed from Figure 15 that when the BP algorithm is implemented with the architecture of this study, the storage resources occupy more than 80%, the logic resources occupy nearly 60%, and the layout and routing results of each part are obtained after comprehensive compilation as Figure 16.

**Figure 17:**
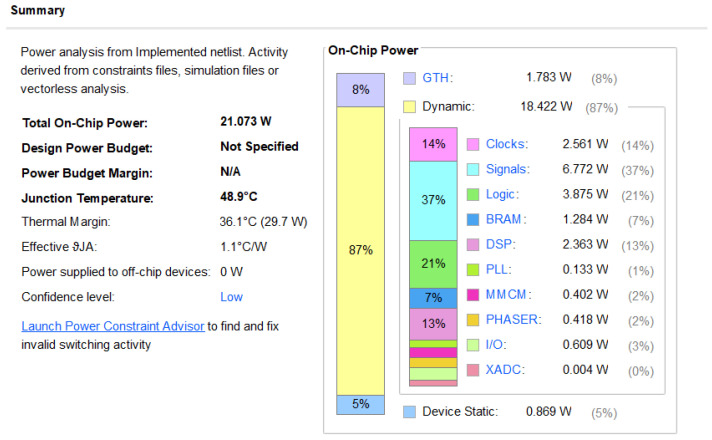
Vivado power analysis.

In conclusion, a single XC7VX690T FPGA chip resource can be fully utilised to complete a 900 × 900 imaging grid. Vivado energy consumption analysis shows that the project has a power consumption of 21.073 W, and the power consumption of each resource was analysed by Vivado as indicated in Figure 17 below.

Based on a review of current literature, it was found that the realisation platform of the BP imaging of SAR radar mainly includes the CPU, DSP, GPU, and FPGA. However, at present, few articles demonstrate the entire process of the BP algorithm with high-temporal effectiveness using FPGA. Based on the analysis and comparison of existing articles, the time consumption of this study is summarised in the following Table 4.

It can be observed from the above table that the use of this architecture to implement the BP algorithm based on FPGA has obvious advantages in terms of time consumption.

**Figure 18:**
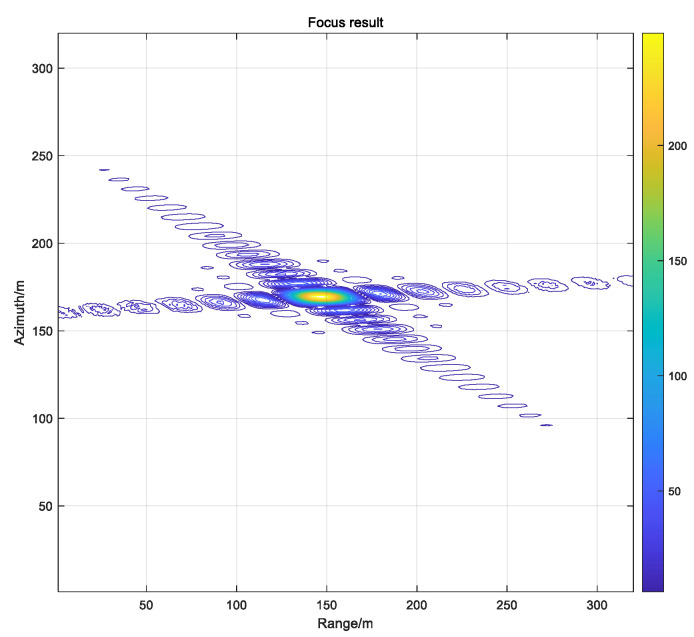
FPGA processing single point results.

In order to verify the imaging performance of the BP algorithm implemented by the FPGA hardware, a test of the target point data generated by the simulation is carried out in squint mode, and the test results are as Figure 18 and Table 5.

From hardware test results on the target point, we see that the BP hardware implementation presented in this paper meets the design expectations.

**Figure 19:**
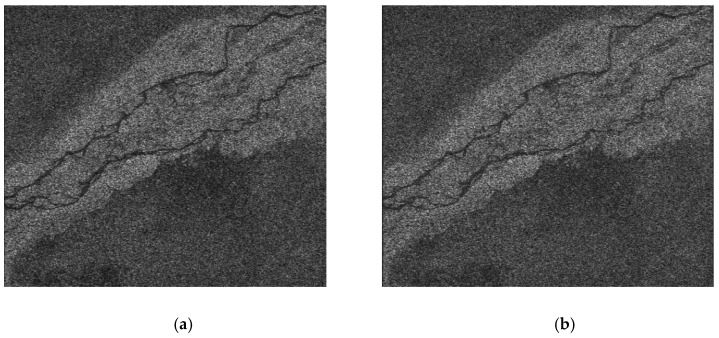
Airborne SAR real-time imaging results. (**a**) Matlab process result. (**b**) FPGA process result.

This real-time imaging technology can be applied to airborne and spaceborne SAR platforms. This study uses airborne SAR radar as an example to process the actual flight data. The image range is 900 pixels and the azimuth is 900 pixels. At the clock frequency of 200 MHz, the processing time is approximately 1.1 s, and the processing result is as shown in the Figure 19 and Table 6.

As observed from the figure above, this method is used to complete imaging processing in a single FPGA, the output image features are distinct, and the focusing effect is obvious.

## 6. Conclusions

This study proposed a real-time BP algorithm implementation method for SAR based on FPGA and deployed it on a single XC7VX690T FPGA chip from Xilinx Inc. Actual flight data were used for validation of the proposed method. The article first introduces the principle of backward projection imaging, analyses the algorithmic flow, operation, and storage requirements; the algorithmic implementation architecture design and optimisation are then conducted, and the optimisation methods of algorithmic flow, fixed-point operation, parallel processing, and distributed storage based on the characteristics of FPGA processor were described in detail. Subsequently, resource utilisation, system power consumption and processing time were analysed based on the hardware platform, and the performance comparison with the existing design reflects the advanced design of the proposed method. Finally, the correctness of the processing architecture is verified by actual flight data. The technology can be used in airborne, spaceborne, and other new platform SAR real-time imaging systems, which can solve the problem of multi-mode, multi-resolution, and multi-geometry signal processing in an integrated way. This study lays the foundation for the subsequent development of high-performance SAR real-time image processing.

## Figures and Tables

**Table 1:** Single point simulation performance index.

Pulse Accumulation Number	Peak Sidelobe Ratio (PSLR) (dB)	Integral Sidelobe Ratio (ISLR) (dB)	Impulse Response 3dB Width (IRW)
5760	−13.20	−10.18	0.93
2880	−13.20	−10.26	1.57
1440	−13.49	−10.36	3.12
720	−13.25	−10.16	6.21
360	−13.26	−10.71	12.4
180	−13.20	−11.92	24.8

**Table 2:** Impact of bit width on processing resources.

Algorithm Module	Fixed Point (10-Bit)	Fixed Point (16-Bit)	Fixed Point (20-Bit)	Single-Precision Floating Point
pulse compression (2048 points)	DSP: 35	DSP: 35	DSP: 35	DSP: 75
	random access memory (RAM): 512 kb	RAM: 512 kb	RAM: 800 kb	RAM: 1600 kb

**Table 3:** FPGA storage resources.

Items	Capability	Order of Magnitude	Bus Width	Bus Speed
register (reg)	<1 M	millions	1 bit	greater than 100 Tbps
static RAM (SRAM)	<100 M	hundreds	1 to 16 bit	less than 1 Tbps
dynamic RAM (DDR)	>1 G	1–4	8 to 64 bit	less than 100 Gbps

**Table 4:** Comparison of processing time consumption.

References and Implementation Platform	Image Size	Processing Time (s)
This research, FPGA @ Xilinx XC7VX690T	900 × 900	1.13
Research [36] Central Processing Unit (CPU) @ Intel i5 3550	1024 × 1024	7256.036
Research [36] GPU @ NVidia GTX 590	1024 × 1024	6.786
Research [37] DSP @ C6678	128 × 4096	9.767
Research [38] Graphics Processing Unit (GPU)	800 × 800	23.695
Research [28] FPGA	512 × 512	3.5

**Table 5:** Result of the single point.

Peak Sidelobe Ratio (PSLR) (dB)	Integral Sidelobe Ratio (ISLR) (dB)	Impulse Response 3dB Width (IRW)
−13.05	−9.21	1.05

**Table 6:** Result of the test image.

	PSNR	SSIM
Image	27.26 dB	0.8652
